# Differential effects of vitamin D on upper and lower body fat-free mass: potential mechanisms

**DOI:** 10.1007/s11033-022-07998-7

**Published:** 2022-11-09

**Authors:** Richard Kirwan

**Affiliations:** Research Institute for Sport and Exercise Sciences, Liverpool John Moores University, Liverpool, UK

**Keywords:** Muscle mass, Mendelian randomization, 25-Hydroxyvitamin D, 25(OH)D, Sarcopenia, Lean mass, Fat-free mass

## Abstract

Vitamin D insufficiency is a global health concern and low vitamin D status is regularly associated with reduced muscle mass and sarcopenia in observational research. Recent research using Mendelian randomization (MR) has highlighted the potentially causal positive effect of serum vitamin D (25(OH)D) on total, trunk and upper body appendicular fat-free mass (FFM). However, no such effect was found in lower body FFM, a result that mirrors the outcomes of some vitamin D intervention studies. Here we review the current literature on vitamin D, muscle mass and strength and discuss some potential mechanisms for the differing effects of vitamin D on upper and lower body FFM. In particular, differences in distribution of the vitamin D receptor as well as androgen receptors, in the upper and lower body musculature, will be discussed.

Low vitamin D status is associated with multiple chronic diseases [1] as well as reduced muscle mass and other aspects of musculoskeletal health [2, 3]. However, up to 40% of the European population is believed to suffer from vitamin D (25(OH) D) insufficiency (25(OH) D level < 50 nmol/L) and while prevalence of deficiency varies by geographical location, ethnicity and multiple other factors, it is widespread enough to be considered a global health issue [4, 5].

Reduced muscle mass and in particular sarcopenia, the age-associated decline in muscle mass, strength and quality [6], are associated with a multitude of chronic conditions. These include cardiovascular disease (CVD) [7], type 2 diabetes mellitus (T2DM) [8], frailty [9], increased risk of falls and fractures [10], cognitive decline and depression [11, 12], and all-cause mortality [13]. Of particular concern, the prevalence of vitamin D deficiency is believed to be particularly high (65%) amongst older adults in the UK [14, 15]. Increased time spent indoors due to poor mobility, also related to impaired muscle function amongst older adults may lead to an even greater risk of vitamin D deficiency [16, 17], which may further contribute to the development of sarcopenia in this already at-risk group.

Recently, our research group published results from Mendelian randomization (MR) analysis which provide evidence for a potentially causal association of serum 25(OH)D with total, trunk and upper body appendicular (arm) fat-free mass (FFM) [18]. These findings are broadly in agreement with some cross-sectional, population-based studies, which have shown a positive relationship between serum vitamin D status and fat free mass [3, 19, 20]. In a population of 127 pre-frail and frail older people (79.0 ± 7.8 y) in the Netherlands, serum vitamin D status was associated with both appendicular lean mass (ALM) (β = 0.012, P = 0.05) and physical performance (β = 0.020, P < 0.05), after adjustment for confounding factors [3]. Similarly, in an adolescent population (15.3 ± 1.9 y) with (60%) and without (40%) type 1 diabetes from Poland, serum vitamin D levels correlated positively with LBM Z-scores (r = 0.3; P = 0.020) [19]. Furthermore, in a meta-analysis of 12 studies with a total of 22,590 subjects, individuals with sarcopenia were reported to have lower blood 25(OH)D concentrations than healthy controls [20].

Additionally, some interventional studies have shown increases in muscle mass upon supplementation with vitamin D [21, 22]. Ceglia et al. investigated the effects of vitamin D supplementation (4000 IU for 4 months) in 21 mobility-limited women (78 ± 5 y). The intervention group (n = 9) was observed to experience a 10.6% ± 20.0% increase in muscle fibre cross-sectional area, compared with the placebo group which experienced a 7.4% ± 18.9% decrease (P = 0.048) [21]. In a study of 77 overweight and obese women (38 ± 8.1 y), 12 weeks of supplementation with vitamin D3 (25 µg per day) lead to a 1.8 ± 2.1 kg increase in FFM in the supplement group compared to a 0.4 ± 2.1 kg increase in the placebo group (p < 0.001) [22].

Indeed, there are a number of potential mechanisms by which vitamin D may exert its effects on skeletal muscle including the regulation of expression of genes involved in muscle growth, via the vitamin D receptor (VDR), as well as non-genomic pathways involved in skeletal muscle intracellular signaling [23]. However, of particular note from our MR analysis was the fact that genetically determined serum 25(OH)D had no statistically significant positive association with lower body appendicular (leg) FFM.

In our paper [18] we acknowledged that some cross-sectional research has revealed similar findings i.e. stronger associations between vitamin D and various measures of muscle mass or strength in the upper compared with lower body appendages [3, 24]. For example, while serum vitamin D status was associated with total ALM (β = 0.012, P = 0.05) in pre-frail and frail older people (79.0 ± 7.8 y), no statistically significant association was observed with leg lean mass alone (β = 0.009, P = 0.079) [3]. Similarly, in a sample of 419 men and women (age range 20–76 y), serum vitamin-D was observed to be associated with both isometric and isokinetic strength of the arms (P < 0.05 for all measures of elbow flexion and extension) [24]. However, serum vitamin-D was only associated with isometric strength of the legs after multivariate regression (P > 0.05 for isokinetic knee extension and flexion) [24]. It should also be noted that studies showing vitamin D supplementation improving lower body strength also exist. In a 6-month trial of vitamin D (90,000-150,000 IU per month) and calcium supplementation, those receiving supplementation experienced an increase in hip flexor strength (16.4% p = 0.0001) and knee extension strength (24.6% p = 0.0007) [25]. The discrepancy in the relationship between leg and arm muscle properties with vitamin-D deserves further consideration and here we elaborate on putative mechanisms.

## Vitamin D receptor and muscle fibre type

Murine cell models have demonstrated that skeletal muscle is a direct target for vitamin D via the VDR, highlighting the importance of vitamin D for muscle hypertrophy [26]. VDR gene knockout (VDR -/-) mice have been observed to have smaller muscle fibre sizes [27, 28] and to have significantly weaker grip strength than controls [28] with similar effects observed due to diet-induced vitamin D deficiency [28]. In rats, overexpression of VDR is known to stimulate muscle hypertrophy through multiple potential mechanisms including increased protein synthesis, translational efficiency, ribosomal expansion and upregulation of genes related to extracellular matrix (ECM) remodeling [29]. As part of the same research study, VDR expression was quantified in human subjects who had performed whole body resistance exercise for 20 weeks and VDR expression was observed to correlate significantly with muscle hypertrophy [29]. Furthermore, it has been proposed that lower vitamin D levels in the elderly may lead to reduced expression of VDR due to downregulation of the receptor [30]. This could potentially reduce protein synthesis leading to a reduction in fat-free mass and specifically, the decline in type 2 fibres characteristic of sarcopenia [31, 32]. Skeletal muscle consists of different muscle fibre types which are broadly classified as type 1 and types 2A, 2X, and 2B, based on different myosin heavy chain (MHC) composition [33]. Indeed, vitamin D supplementation has been shown to activate VDR in skeletal muscle tissue, stimulating protein synthesis [34] and to increase the size and number of type 2A muscle fibres in older adults when supplemented daily with calcium for 3–6 months [35]. in vitamin D-insufficient women, Ceglia et al. [21] reported that 4 months of daily vitamin D3 (4000IU), lead to a 10% increase in muscle fibre size and a 30% increase in intramyonuclear VDR concentration, particularly in type 2 muscle fibres [21]. However, there was no difference between supplemented and control groups in measures of knee extension strength and short physical performance battery score, nor were there any measures of muscle mass [21].

Fibre types are known to be differentially distributed in skeletal muscle throughout the body [36]. For example biceps brachii have been reported to contain a higher proportion of type 2 fibres [37], while the knee extensors are known to have higher proportion of type 1 fibres [38]. Broadly speaking, upper body appendicular skeletal muscle has a higher proportion of type 2 fibres with a higher proportion of type 1 fibres found in human lower body appendicular muscle [36, 39]. A recent study by Srikuea et al. reported higher levels of the VDR in predominantly type 2 muscles (plantaris) compared to predominantly type 1 muscles (soleus) in a mouse model [40]. These findings may suggest that a factor related to fibre type composition may contribute to VDR expression in different muscles. Interestingly, in a rat model of VDR-overexpression, type 2x fibres displayed a greater content of satellite cells per fibre than controls (0.024 ± 0.007 per fibre vs. 0.014 ± 0.006 per fibre, respectively, P < 0.05) [29]. Satellite cell accumulation is believed to contribute to the hypertrophic response to resistance exercise [41].

One could speculate that the greater density of VDR in type 2 muscle fibres, found in proportionally greater quantity in upper body appendicular muscle compared to lower body, may lead to the positive effect of vitamin D status on arm fat-free mass observed in our MR analysis.

## Testosterone and androgen receptors

Other potential causes for discrepancies in the effect of vitamin D on upper and lower appendicular muscles should also be considered. For example, another difference between upper and lower body musculature is the content of androgen receptors (AR) which have been reported to be higher in upper-body muscles (trapezius) compared with lower body muscles (vastus lateralis) [42]. It is feasible to imagine that this difference in ARs might lead to differential effects of androgen hormones such as testosterone in upper and lower body skeletal muscle. Vitamin D is thought to play a role in the development and expression of genes in the testes [43], including the production of sex-steroid hormones such as testosterone [44]. Research using isolated human adult primary testicular cells has revealed that treatment with vitamin D3 results in an upregulation of enzymes involved in androgen production as well as an increase in testosterone synthesis [44].

**Fig. 1:**
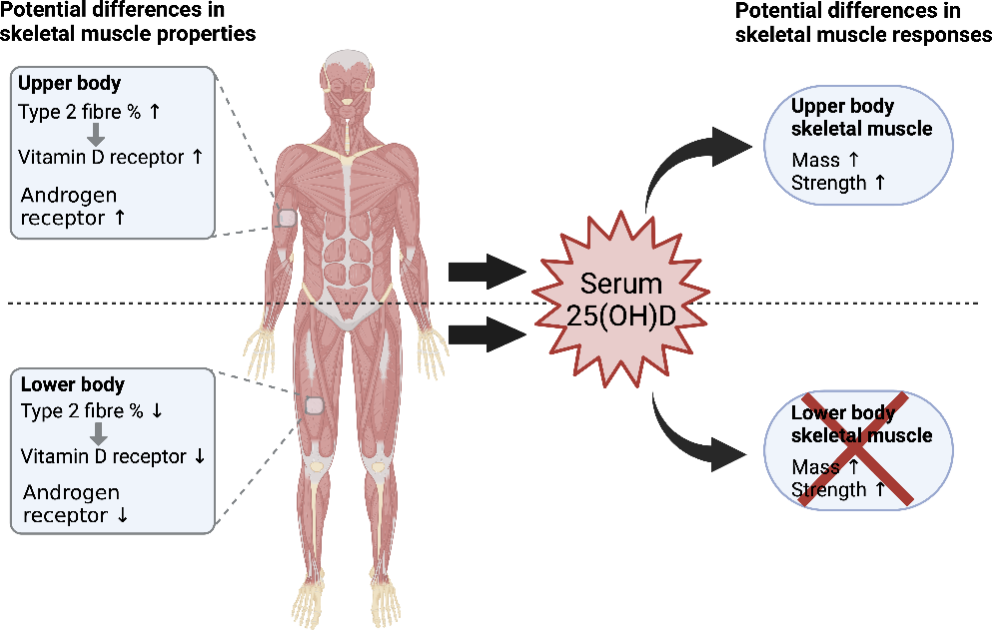
Potential mechanisms for differential upper and lower body muscle property responses to increased serum 25(OH)D levels. Upper body skeletal muscle contains a proportionally greater proportion of type 2 muscle fibres which are known to have a higher concentration of the vitamin D receptor. Vitamin D is also associated with elevated testosterone levels which interact with skeletal muscle androgen receptors, also higher in upper body skeletal muscle. Created with BioRender.com

In men, higher serum 25(OH)D (≥ 75 nmol/L) has also been associated with significantly higher levels of testosterone [45–47], an androgen hormone associated with muscle mass, and Free Androgen Index (FAI) compared to those with lower levels [46]. However, some cross-sectional research has found no such association [48] or even a negative association between serum vitamin D levels and circulating free testosterone levels [49]. Furthermore, in a trial of 54 men (mean age 49.4 years) with insufficient vitamin D levels (< 50 nmol/L), daily supplementation of vitamin D (3,332 IU) for 1 year lead to significant increases in both total and free testosterone [50]. It could be speculated that the testosterone-stimulating effects of vitamin D may have a greater effect on upper body lean mass due to a potentially greater content of androgen receptors in upper body musculature [42]. Figure 1 illustrates the potential VDR and androgen receptor dfferences of upper and lower body skeletal muscle in response to serum 25(OH)D.

## Metabolic mechanisms

Additionally, there are a number of metabolic differences between these upper and lower body muscle groups, particularly related to fat utilization. Specifically, compared to legs, arm muscles have been reported to display lower fat oxidation capacity [51], lower 3-hydroxy-acyl-CoA-dehydrogenase (HAD) activity (necessary for fatty acid oxidation) [39], lower intramyocellular lipid (IMCL) content [52], and higher exercise-induced lactate release [53]. While these factors are not directly related to fat-free mass, they highlight some considerable differences in upper and lower body skeletal muscle metabolism, which could have implications for the effects of vitamin D status on muscle size in the upper and lower extremities.

A growing body of literature highlights the importance of serum vitamin D levels in muscle mass hypertrophy and maintenance i.e. body compositional changes that are associated with lower morbidity and mortality rates [7–13]. As the aging process is known to decrease the amount of vitamin D produced in the skin [54], the elderly, a population already at considerable risk of sarcopenia [6], may be even more likely to suffer muscle loss due, in part, to vitamin D deficiency. Considering the globally widespread prevalence of vitamin D insufficiency [4, 5], methods for attaining and maintaining sufficient serum 25(OH)D levels in the population deserve investigation and implementation. Furthermore, current research highlights that lower leg muscle function in particular is associated with greater risk of some chronic diseases [55]. If it is indeed the case that vitamin D is of less benefit to lower body muscle mass and strength, then some vitamin-D-related strategies to prevent or ameliorate sarcopenia may need to be reconsidered. The discrepancies in the effects of vitamin D on arm and leg muscle mass and strength, reported in our recent MR analysis and in observational and intervention studies warrant further investigation to better understand their potential mechanisms.

## Data Availability

Not applicable.
